# The structure and mechanism of action of a distinct class of dicistrovirus intergenic region IRESs

**DOI:** 10.1093/nar/gkad569

**Published:** 2023-07-10

**Authors:** Irina S Abaeva, Christina Young, Reid Warsaba, Nadiyah Khan, Lan Vy Tran, Eric Jan, Tatyana V Pestova, Christopher U T Hellen

**Affiliations:** Department of Cell Biology, SUNY Downstate Health Sciences University, Brooklyn, NY 11203, USA; Department of Biochemistry and Molecular Biology, Life Sciences Institute, University of British Columbia, Vancouver, BC V6T 1Z3, Canada; Department of Biochemistry and Molecular Biology, Life Sciences Institute, University of British Columbia, Vancouver, BC V6T 1Z3, Canada; Department of Biochemistry and Molecular Biology, Life Sciences Institute, University of British Columbia, Vancouver, BC V6T 1Z3, Canada; Department of Biochemistry and Molecular Biology, Life Sciences Institute, University of British Columbia, Vancouver, BC V6T 1Z3, Canada; Department of Biochemistry and Molecular Biology, Life Sciences Institute, University of British Columbia, Vancouver, BC V6T 1Z3, Canada; Department of Cell Biology, SUNY Downstate Health Sciences University, Brooklyn, NY 11203, USA; Department of Cell Biology, SUNY Downstate Health Sciences University, Brooklyn, NY 11203, USA

## Abstract

Internal ribosomal entry sites (IRESs) engage with the eukaryotic translation apparatus to promote end-independent initiation. We identified a conserved class of ∼150 nt long intergenic region (IGR) IRESs in dicistrovirus genomes derived from members of the phyla *Arthropoda, Bryozoa, Cnidaria, Echinodermata, Entoprocta, Mollusca* and *Porifera*. These IRESs, exemplified by Wenling picorna-like virus 2, resemble the canonical cricket paralysis virus (CrPV) IGR IRES in comprising two nested pseudoknots (PKII/PKIII) and a 3′-terminal pseudoknot (PKI) that mimics a tRNA anticodon stem–loop base-paired to mRNA. However, they are ∼50 nt shorter than CrPV-like IRESs, and PKIII is an H-type pseudoknot that lacks the SLIV and SLV stem–loops that are primarily responsible for the affinity of CrPV-like IRESs for the 40S ribosomal subunit and that restrict initial binding of PKI to its aminoacyl (A) site. Wenling-class IRESs bound strongly to 80S ribosomes but only weakly to 40S subunits. Whereas CrPV-like IRESs must be translocated from the A site to the peptidyl (P) site by elongation factor 2 for elongation to commence, Wenling-class IRESs bound directly to the P site of 80S ribosomes, and decoding begins without a prior translocation step. A chimeric CrPV clone containing a Wenling-class IRES was infectious, confirming that the IRES functioned in cells.

## Graphical Abstract

**Graphical Abstract:**
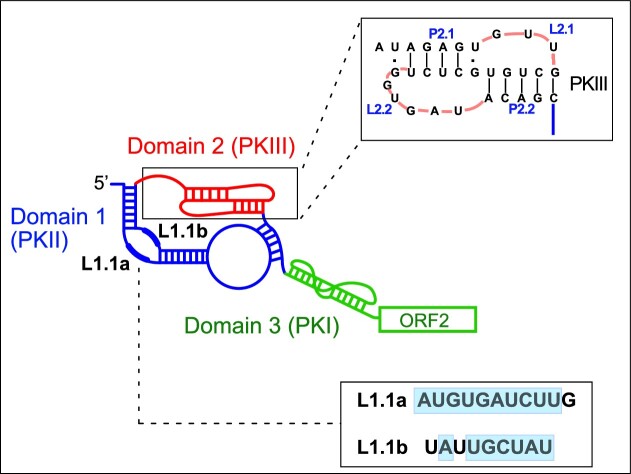

## INTRODUCTION

Internal ribosomal entry sites (IRESs) are complex, highly structured RNA elements that are mostly located in the 5′-untranslated regions (UTRs) of mRNAs and that promote end-independent initiation of translation by binding in a non-canonical manner to canonical components of the translation apparatus (1–3). Their presence in viral mRNAs enables synthesis of viral proteins to persist during infection when cellular translation is down-regulated by the activated innate immune response and by virus-mediated modification of the translation apparatus (1). There are several major classes of viral IRES, each of which has a conserved structure and sequence motifs, and each of which utilizes a specific subset of eukaryotic initiation factors (eIFs) to promote initiation (1–3).

The smallest, most compact IRESs occur in the intergenic region (IGR) of the single-stranded, positive-sense RNA genomes of dicistroviruses, between open reading frame 1 (ORF1) which encodes a picornavirus-like helicase–protease–polymerase polyprotein, and ORF2, which encodes capsid proteins. Dicistroviruses are accordingly classified as members of the ‘Picorna-Calici clade’. We suggested that dicistrovirus IGR IRESs be designated as type 6 (4,5). They form two major classes: type 6a, which is 176–193 nt long and is epitomized by cricket paralysis virus (CrPV), and type 6b, which is 195–205 nt long and is epitomized by Taura syndrome virus (TSV) (6). They both have a 3′-terminal pseudoknot (PKI) immediately upstream of the first codon of ORF2 (which is commonly a GCU, GCA or CAA triplet) and a 5′-terminal domain formed by nested PKII and PKIII pseudoknots. PKI mimics the anticodon stem–loop of a tRNA base-paired to mRNA, PKII contains an internal loop (L1.1) that binds to the L1 stalk of the large (60S) ribosomal subunit, and PKIII contains conserved stem–loops IV (SLIV) and SLV that bind to ribosomal proteins in the head of the 40S ribosomal subunit (7–9). Type 6b IRESs contain an additional hairpin in PKI (10–12). IGR IRESs utilize the most streamlined mechanism of initiation, which does not involve an initiation codon, initiator tRNA or initiation factors (13–16). Instead, they either bind to a 40S subunit and then join with a 60S subunit to form an 80S ribosome or bind directly in the ribosomal intersubunit space. Binding affinity is largely determined by SLIV and SLV in PKIII (17). PKI initially occupies the ribosomal A site and must undergo elongation factor 2 (eEF2)-mediated translocation to the P site to enable eEF1A-GTP/aminoacyl-tRNA to bind to the A site (9,18,19). Elongation commences after the aminoacyl-tRNA has been released from eEF1A and translocated to the P site by eEF2. Type 6 IRESs therefore bypass the conventional initiation process completely and instead mediate the recruitment of ribosomes to the first codon of ORF2, and their direct progression to the elongation stage of translation.

Type 6 IGR IRESs are remarkable in that although they are only ∼200 nt long, they mimic mRNA and tRNA, interact specifically with 40S and 60S subunits and determine the start site for translation. We recently identified a group of even shorter IGR IRESs (‘type 6c’), epitomized by Halastavi árva virus (HalV) that contain PKI and PKIII elements but lack PKII (5), and another group (‘type 6d’) epitomized by Nedicistrovirus (NediV) that has a divergent PKII domain that lacks SLV (20). These structural differences suggest that IRES types 6a, 6b and 6d may have evolved from ancestral type 6c-like IRESs by accretion of subdomains. Recent metagenomic studies have led to enormous increases in the known extent of the virosphere. The Picorna-Calici clade contains a large number of novel viruses [e.g. (21–25)] and is thought to have originated and diversified early in eukaryotic evolution (26). Here, we report that analysis of metagenomic datasets derived from eukaryotic marine and freshwater organisms revealed a group of dicistrovirus IGR IRESs that is structurally distinct from established types of IGR IRESs, and that biochemical analysis characterized the mechanism by which its members mediate initiation of translation.

## MATERIALS AND METHODS

### Sequences

Sequences were analyzed from viruses as indicated in the text (name followed by accession number) and from halhan virus 1 (MG210797); Beihai picorna-like virus 85 (KX883346); bivalve RNA virus G1 (NC_032112.1); bivalve RNA virus G5 (NC_032115); bivalve RNA virus G5 strain Abbotsbury/A/2016 (MW588039.1); Caledonia beadlet anemone dicistro-like virus 1 (MF189971.1); picornavirales N_OV_008 (KY130493), Q_sR_OV_023 (KY286103.1), Q_sR_OV_042 (KY286099.1) and sp. isolate HPLV-11 (OM622266.1); Wenling picorna-like virus 2 (WLJQ104117); Wenling crustacean virus 3 (KX884321.1) and from Transcriptome Shotgun Assembly (TSA) sequences from *Anemonia viridis* (GHCD01039554.1); *Eusirus giganteus* (GIYD01004667.1); *Halisarca dujardinii* (*GIFI01359033.1*);*Limacina antarctica* (GDRM01060271.1, GDRM01080810.1); *Loxechinus albus* (GGVM01178958); *Loxomitra* sp. KK-2020 (GIMU01060471.1); *Octopus vulgaris* (GKAX01229880.1); *Oratosquilla oratoria* (GHJD01025978.1); *Penaeus semisulcatus* (GGTV01000394.1); *Proasellus karamani* (HAFC01081091.1); *P. spelaeus* (HAFK01011955.1); *P. solanasi* 2 (HAFJ01050538.1); and *Terminoflustra membranaceotruncata* (GIMX01014423.1).

### Identification of candidate IGR IRESs

Candidate IGR IRES sequences were identified in the NCBI database using BLASTN (http://www.ncbi.nlm.nih.gov/BLAST/) to search nucleotide collection (nr/nt) and TSA sequences, and BLASTX to search non-redundant protein and TSA protein sequences. Nucleotide and polypeptide searches used the parameters: E, 1000; word size, 11; match/mismatch scores, 1/1; gap costs, 2/1; and E, 1000; word size, 6; Matrix: BLOSUM62; gap costs, 9/1, respectively. Hits were characterized by six-frame translation (http://molbiol.ru/eng/scripts/01_13.html) to identify those sequences that corresponded to genomic fragments of members of the *Picornavirales*. Sequences of potential translation products were used in BLAST searches to verify that the C-terminal region of ORF1 encoded the 3D polymerase and that ORF2 encoded capsid proteins; Clustal Omega (http://www.ebi.ac.uk/Tools/msa/clustalo/) was used to align ORF2 and known dicistrovirus ORF2 sequences to identify potential initiation codons and, with ORF1 sequences, to estimate the borders of potential IRESs. IGR sequences were aligned using Clustal Omega and EMBOSS Matcher (http://www.ebi.ac.uk/Tools/psa/emboss_matcher/nucleotide.html). Clustal Omega was used to determine identities between viral 3CD and capsid protein sequences.

### Phylogenetic analysis

Dicistrovirus ORF2 amino acid sequences were submitted to phylogeny.lirmm.fr (27) for alignment using CLUSTAL-W (default parameters) and elimination of positions containing gaps, and then used to infer the maximum likelihood phylogenetic tree using IQ-TREE (28) applying the LG + F + R5 model for amino acid substitution as the best predicted model (29) and using 1000 ultrafast bootstraps to estimate the statistical support for individual nodes (30). Trees were visualized with iTOL (31).

### Modeling of IGR IRES structures

Secondary structures were identified by free energy minimization using Mfold (32). Tertiary structures were initially modeled using pKiss (http://bibiserv2.cebitec.uni-bielefeld.de/pkiss) (33) and IPKnot (http://rtips.dna.bio.keio.ac.jp/ipknot/) (34) with default parameters. Mutational analysis was undertaken to test structural models and to resolve the structure of regions of ambiguity. The quality of models was assessed by determining their compatibility with sequence variation between viruses.

### Plasmids

Transcription vectors for tRNA^Ser^ (UCU codon) and tRNA^Ala^ (GCU codon) have been described (35,36). Transcription vectors for wild-type and mutant IGR-containing mRNAs contained a T7 promoter, the sequence 5′-GGCCGACCCGGTGACGGGTCGGCC-3′ [to form a stable hairpin (Δ*G* = –25.80 kcal/mol)] and the viral sequence of interest with a common primer-binding site (5′-CAAGGCAATCACACC-3′) replacing viral sequences 61–75 nt downstream of the putative initiation codon, cloned between BamHI and XbaI sites of pUC57 (Genwiz, South Plainfield, NJ, USA). Viral sequences corresponded to Beihai picorna-like virus 85 nucleotides 5586–5870, bivalve RNA virus G1 nucleotides 6331–6610, bivalve RNA virus G5 nucleotides 4590–4880, Caledonia beadlet anemone dicistro-like virus 1 nucleotides 6338–6624, *L. antarctica* TSA1 nucleotides 865–1150*, *L. antarctica* TSA2 nucleotides 641–937*, picornavirales N_OV_008 nucleotides 4786–5071, Q_sR_OV_023 nucleotides 5896–6182, Q_sR_OV_042 nucleotides 6651–6941, *P. karamani* TSA nucleotides 101–386*, *P. solanasi* TSA2 nucleotides 1021–1303, *P. spelaeus* TSA nucleotides 1878–2165, Wenling picorna-like virus 2 nucleotides 6729–7013 and Wenling crustacean virus 3 nucleotides 48–346 (* indicates numbering of the antiparallel sequence). mRNAs and tRNAs were transcribed using T7 RNA polymerase. tRNA^Ala^ was refolded as described (14). Vectors for expression in *Escherichia coli* of eRF1 and eRF3 (amino acids 139–499) (referred to here as eRF3) have been described (5,37). The vector pET15b-His_6_-SERBP1 for expression in *E. coli* of N-terminally His_6_-tagged human SERBP1 (NCBI Ref. seq. NM_015640.4) was made by inserting appropriate DNA between NdeI/BamHI sites of pET15b (GenScript, Piscataway, NJ, USA).

The chimeric infectious CrPV/Caledonia clone was made by replacing the CrPV IGR IRES by Caledonia nucleotides 6368–6514 as described (38). The substitutions C775T and G778T generated a stop codon in ORF1 of the wild-type pCrPV-3 sequence (GenBank: KP974707.1) (38), yielding CrPV-(ORF1 Stop). The substitution A6232T made in ORF2 of the wild type and hybrid clones yielded CrPV-(ORF2 Stop) and CrPV–Caledonia-(ORF2 Stop). Disruption of base pairing in Caledonia PKI by GTC5908-5910CAG substitutions yielded CrPV–Caledonia-(ΔPKI). All plasmids were sequence verified by Plasmidsaurus (Eugene, OR, USA). Plasmids containing CrPV and chimeric CrPV–Caledonia infectious clones were linearized with Eco53KI. RNA was transcribed using T7 RNA polymerase, then purified using an RNeasy kit (Qiagen). RNA integrity was confirmed on a 1% denaturing formaldehyde agarose gel.

### Purification of factors and ribosomal subunits, and aminoacylation of tRNA

The 40S and 60S ribosomal subunits, eEF1H, eEF2 and total (Σ) aminoacyl-tRNA synthetases were purified from rabbit reticulocyte lysate (Green Hectares, Oregon, WI, USA) (14,39,40). Ribosomes from *Spodoptera frugiperda* Sf21 cell extracts (Promega) were purified using the same protocol. eRF1 and eRF3 were expressed and purified (5). RelE (41) was a gift from V. Ramakrishnan (MRC LMB, Cambridge, UK). Native total calf liver tRNA (Promega), and *in vitro* transcribed tRNA^Ala^ and tRNA^Ser^ were aminoacylated using total native aminoacyl-tRNA synthetases (40,42). Recombinant His_6_-tagged Serbp1 was expressed and purified as described (35).

### Assembly and analysis of ribosomal complexes

For toeprinting analysis, ribosomal complexes were assembled essentially as described (14) unless otherwise stated in the text. Aliquots of 2 pmol of IGR-IRES RNA were incubated for 5 min at 37°C in 40 μl reaction volumes that contained buffer A [2 mM dithiothreitol (DTT), 100 mM potassium acetate, 20 mM Tris (pH 7.5), 2.5 mM magnesium acetate, 1 mM ATP, 0.1 mM GTP and 0.25 mM spermidine] and 6 pmol 40S subunits with or without 8 pmol 60S subunits, 20 pmol eRF3 and 15 pmol eRF1 as indicated.

To examine the elongation competence of 80S complexes, reaction mixtures were supplemented with combinations of 10 pmol eEF2, 50 pmol eEF1H, 15 μg of aminoacylated native total tRNA (Σaa-tRNA), Ser-tRNA^Ser^ or Ala-tRNA^Ala^, and 500 μg/ml cycloheximide, and incubation was continued for another 10 min. The resulting complexes were analyzed by primer extension using avian myeloblastosis virus (AMV) reverse transcriptase (Promega) and a primer (5′-GGTGTGATTGCCTTG) complementary to the common primer-binding site 61–75 nt downstream of each putative start codon that had been 3′-[^32^P]-end-labeled (39). cDNA products were resolved in 6% polyacrylamide sequencing gels, followed by autoradiography, and were compared with appropriate dideoxynucleotide sequence ladders to map the positions of toeprints.

The influence of Serbp1 and eEF2 on ribosomal binding was assayed by incubating 1 pmol mRNA for 5 min at 37°C in a 40 μl reaction volume containing buffer A and 3 pmol 40S subunits, 4.5 pmol 60S subunits or both, 8 pmol eEF2 and 8 pmol SERBP1. Toeprinting was done as above. cDNA products were resolved in 6% polyacrylamide sequencing gels, followed by visualization using a Typhoon biomolecular imager (GE Healthcare).

### Analysis of ribosomal complexes by RelE cleavage

Cleavage of ribosome-bound mRNA by RelE was analyzed as described (43). The 80S ribosomal complexes were assembled with or without eEF2 as described above for analysis of elongation competence and then incubated with 20 pmol RelE for 10 min at 37°C. mRNA was phenol extracted and analyzed by primer extension using AMV RT and the same ^32^P-labeled primers as those used for toeprinting analysis. cDNA products were resolved in 6% polyacrylamide sequencing gels.

### Chemical and enzymatic probing

A 5 pmol aliquot of *P. solanasi* TSA2 mRNA nucleotides 1021–1303 was digested with RNase T1 (0.04 U/μl) or modified by incubation with 1-cyclohexyl-(2-morpholinoethyl) carbodiimide metho-*p*-toluene sulfonate (CMCT) (12.6 mg/ml) for 10 min at 30°C in 50 μl of buffer A (20 mM Tris pH 7.5, 100 mM KCl, 1 mM DTT, 2.5 mM MgCl_2_, 0.25 mM spermidine) (44) or with 2-methylnicotinic acid imidazolide (NAI) in dimethylsulfoxide (DMSO) for 30 min at 37°C (45). Cleaved or modified sites were identified by primer extension.

### Cell culture

*Drosophila* Schneider line 2 (S2) cells were maintained and passaged at 25°C in Schneider's insect medium (Sigma-Aldrich) supplemented with 10% fetal bovine serum and 5% penicillin–streptomycin. Transfection of *in vitro* synthesized RNA (2  μg) into 2.5 × 10^6^ S2 cells was performed using Lipofectamine 2000 (Invitrogen).

### Virus infection and titer

*Drosophila* S2 cells were infected with CrPV and Caledonia–CrPV chimeric viruses at the indicated multiplicity of infection (MOI) in phosphate-buffered saline (PBS). After 30 min, cells were supplemented with complete medium and incubated at 25°C.

Viral titers were determined as described (38). Briefly, infected cells were incubated for 8 h before fixing in 3% paraformaldehyde and permeabilization in methanol. Cells were probed by rabbit α-CrPV VP2 (1:500, Genscript) in 5% bovine serum albumin (BSA) in PBS, and then with secondary goat α-rabbit Texas Red IgG (1:200; Life Technologies) in 5% BSA solution. Nuclei were stained with Hoechst dye (0.5 μg/ml; Life Technologies). Infected cells were visualized using an EVOS FLoid imaging system and quantified using ImageJ [fluorescent-forming units (FFU)/μl].

### Western blot

Equal amounts of S2 protein lysates were resolved on a 15% sodium dodecylsulfate (SDS)–polyacrylamide gel electrophoresis (PAGE) gel and transferred onto a polyvinylidene difluoride Immobilon-FL membrane (Millipore). Membranes were blocked for 1 h at room temperature in 5% skim milk in 50 mM Tris, 150 mM NaCl, 0.1% Tween 20, pH 7.4. Blots were probed with rabbit polyclonal antibody against CrPV ORF2 (VP2) (1:1000, Genscript) or mouse anti-S2 Tubulin (1:1000; Santa Cruz). Membranes were then incubated for 1 h in goat α-rabbit Texas Red (1:3000, Invitrogen) or goat α-mouse Alexa Fluor 488 (1:3000, Invitrogen). Fluorescence was detected by Typhoon imager (Amersham).

### Quantification and statistical analysis

All *in vitro* experiments were repeated at least three times, and they included technical and biological replicates. Representative gel images and sucrose density gradient graphs are shown.

## RESULTS

### Identification of a novel class of dicistroviruses

Database searches to identify Halastavi árva virus (HalV)-like IGR IRESs (5) yielded candidates with HalV-like IGR sequence motifs that were 13–28 nt longer than members of the Halárvirus clade. Some candidates occur in uncategorized viral genomes, including Beihai picorna-like virus 85 (Beihai 85), Wenling picorna-like virus 2 (Wenling 2) and Wenling crustacean virus 3 (Wenling 3) (21), bivalve RNA viruses G1 and G5 (bivalve G1 and G5) (46), Caledonia beadlet anemone dicistro-like virus 1 (Caledonia) (23), picornavirales N_OV_008, Q_sR_OV_023, Q_sR_OV_042, HPLV-11 (25,47) and halhan virus 1. Others occur in TSA sequences from the sea snail *Limacina antarctica* (48), the amphipod crustacean *Eusius giganteus* (49), the isopod crustaceans *Proasellus spelaeus, P. solanasi* and *P. karamani* (50), the sea anemone *Anemonia viridis* (51), the sea urchin *Loxechinus albus* (52), the bryozoan *Terminoflustra membranaceotruncata*, the entroproct *Loxomitra* sp. KK-2020 (53), the green tiger prawn *Penaeus semisulcatus* (54), the mantis shrimp *Oratosquilla oratoria*, the common octopus *Octopus vulgaris* (55) and the sponge *Halisarca dujardinii* (56). We refer to these sequences as *A. viridis* TSA, *E. giganteus* TSA, *H. dujardiniii TSA*,*O. oratoria* TSA, *O. vulgaris* TSA, *L*. sp. KK-2020 TSA, *L. albus* TSA, *L. antarctica* TSA1, *L. antarctica* TSA2, *P. semisulcatus, P. karamani* TSA, *P. spelaeus* TSA, *P. solanasi* TSA2 and *T. membranaceotruncata* TSA. They were derived from organisms belonging to seven metazoan phyla: *Arthropoda, Bryozoa, Cnidaria, Echinodermata, Entoprocta, Mollusca* and *Porifera*.

Sequences were identified as fragments of dicistrovirus-like genomes because the IGR was flanked by complete or partial ORFs that encode dicistrovirus-like non-structural and structural polyproteins, respectively. The capsid proteins are encoded in the order VP2–VP4–VP3–VP1 that is characteristic of dicistroviruses; they contain conserved amino acid residues that stabilize ‘domain swapping’ of the N-terminal region of VP2 and the conserved DDF/DDY motif in VP1 that is implicated in catalyzing VP4–VP3 cleavage (57,58). These properties differentiate dicistroviruses from other families in *Picornavirales*.

**Figure 1.**
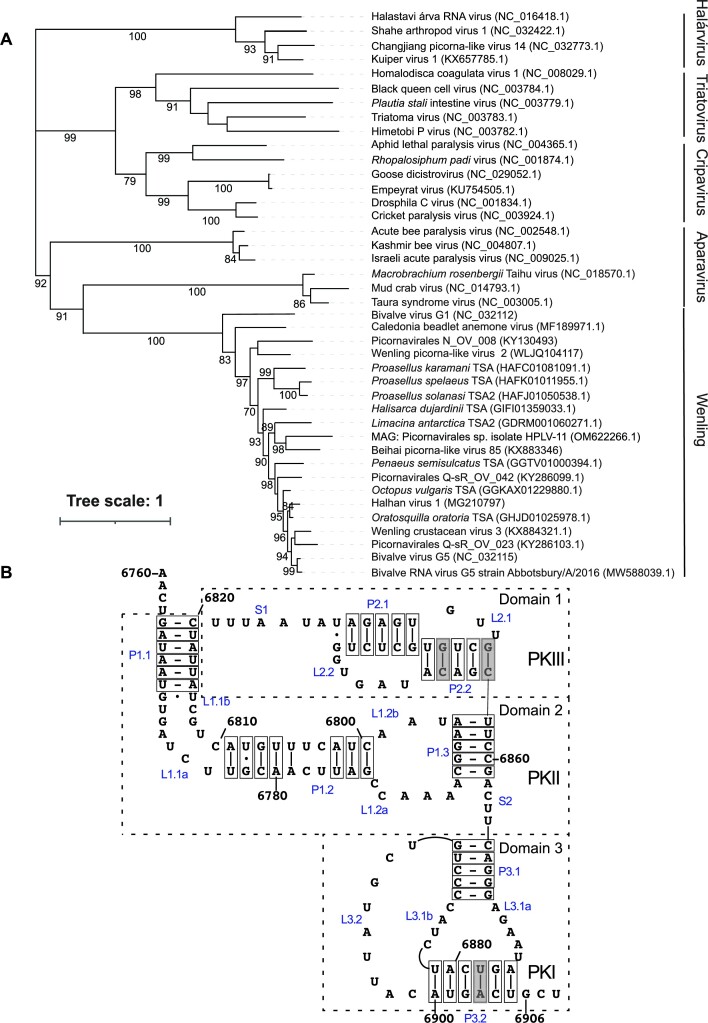
Coherence of the Wenling group of viral genomes. (**A**) Phylogenetic analysis of the ORF2 capsid protein precursors from the indicated viruses, showing that the members of the Wenling group belong to a clade that is distinct from the Halárvirus clade, the *Cripavirus* genus, the *Triatovirus* genus and the two clades in the *Aparavirus* genus of *Dicistroviridae*. The numbers at the branch nodes represent the bootstrap confidence level (above 70). Bar, one amino acid substitution per site. Accession numbers are indicated in parentheses. (**B**) Model of the Wenling 2 IGR IRES structure, annotated to show helices (P), loops (L), single-stranded elements (S), pseudoknots (PK) I, II and III, and domains 1–3. Nucleotides are numbered at 20 nt intervals. The GCU_6906–6908_ triplet adjacent to PKI is the first codon in ORF2. Base pairs that are formed by compensatory substitutions in ≥22 members are boxed; base pairs that are conserved in all 26 members of the group are shaded and boxed.

Some sequences do not correspond to complete viral genomes, precluding comprehensive phylogenetic characterization, but analysis of the capsid-coding region from a subset of these viruses indicated that they form a phylogenetically coherent group that is distinct from established dicistrovirus genera and members of the Halárvirus clade (Figure 1A). Analysis of 2C helicase and 3D polymerase sequences in subsets of these genomes has led to similar conclusions (25,59–61). Amino acid sequence identity in ORF1 (3C protease and 3D polymerase) and ORF2 (capsid proteins) for the analyzed viruses is in the range of 48–96% and 46–93%, respectively (Supplementary Tables S1 and S2). In contrast, amino acid sequence identity between these and corresponding regions in representative Halárvirus group genomes did not exceed 28% and 22% for the 3CD and P1 regions, respectively. Identity between these and corresponding regions of members of the *Cripavirus* genus (CrPV), *Triatovirus* genus (Triatoma virus) and the two divergent clades of insect- and crustacean-infecting viruses (ABPV and TSV, respectively) in the *Aparavirus* genus of *Dicistroviridae* (www.ictv.global/report/dicistroviridae) (62) did not exceed 33% for the 3CD region and 27% for the P1 region, respectively. The ‘Wenling’ viruses therefore form a coherent group and may constitute a distinct genus of *Dicistroviridae*.

### Sequence and structural homogeneity of type 6e IGR IRESs

The putative intergenic IRESs of this distinct clade of *Picornaviridae* are 142–154 nt long, including the first codon of ORF2, which in different IRESs is GCU (Ala), ACU or ACC (Thr), or UCU, UCA or UCG (Ser). These IRESs, here designated type 6e, are thus intermediate in length between type 6c IGR IRESs (126–132 nt) on the one hand and type 6d (161–168 nt), type 6a (176–193 nt) and type 6b IGR IRESs (195–205 nt) on the other. The compact structure of type 6e IRESs comprises two domains (domain 1/PKII and domain 3/PKI) that are analogous to domains 1 and 3 in type 6a, 6b, 6c and 6d IGR IRESs (5,20,63), and a conserved, type 6e-specific pseudoknot that occurs at the same location as domain 2/PKIII of type 6a and 6b IRESs (Figure 1B; Table 1), but that is smaller and lacks elements analogous to SLIV and SLV. Differences in the lengths of type 6e IRESs are primarily limited to insertions/deletions in the L1.2a, L1.2b and S1 loops.

**Table 1.** Conserved sequence motifs in L1.1a and L1.1b loops and PKIII of type 6e IGR IRESs

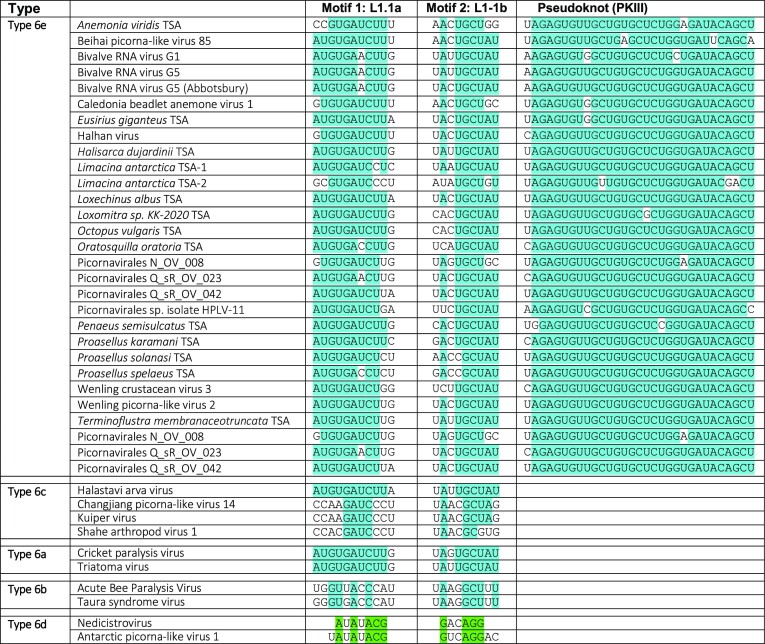

Residues conserved in IGR IRES types 6A, 6b, 6c and 6e are highlighted in blue, and residues conserved in type 6d IGR IRESs (20) are highlighted in green. Type 6c IGR IRESs lack PKIII, and the pseudoknot PKIII sequences in type 6a, type 6b and type 6d IRESs are not shown because they ae unrelated to those in type 6b IRESs.

The Wenling-2 IRES (Figure 1B) is representative of type 6e IRESs. It contains 41 base pairs, 4 of which are invariant in all group members identified to date; the others are invariant or covariant in ≥22 group members, thus ensuring a common pattern of folding of individual helices and of the global structure. The observation that a G·U wobble base pair at the junction of P1 and L1 elements is invariant may point to a function for it in stabilization of IRES structure or in involvement in an RNA–protein interaction (64). Structural heterogeneity is greatest in helix P1.2, which contains 11 base pairs in Picornavirales N_OV_008 but only 5 in *L. antarctica* TSA2, which has an enlarged loop L1.2.

Pairwise identity between type 6e IRESs is high (58–95%) (Supplementary Table S3), and almost 30% of nucleotides are invariant in all 26 members of the group. Sequence conservation is greatest in PKIII, which is specific to type 6e IRESs, and in loops L1.1a and L1.1b, which closely resemble corresponding elements in type 6a, 6b and 6c but not in type 6d IGR IRES (5,6,20) (Table 1).

### Activity of the Caledonia beadlet anemone dicistro-like virus 1 IGR IRES in cultured *Drosophila* cells

Analysis of type 6e IRES function in the context of cognate viral genomes is not possible because appropriate infectious clones have not been established. However, chimeric clones can be generated using the CrPV infectious clone (38), enabling the function of heterologous IGR IRESs and their ability to support viral replication and infection to be characterized in cultured cells (65).

**Figure 2.**
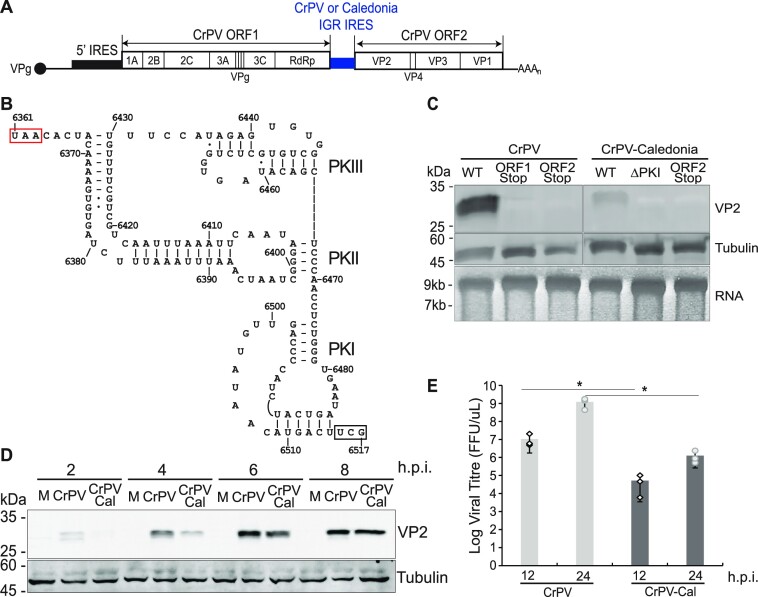
Chimeric CrPV infectious clone containing the Caledonia IGR IRES supports viral replication. (**A**) Schematic of the CrPV genome derived from an infectious clone containing either the CrPV or the Caledonia IRES. (**B**) Model of the Caledonia IGR IRES, labeled to show pseudoknots (PKs) I, II and III, the ORF1 stop codon (red box) and the ORF2 start codon (black box). Nucleotides are numbered at 10 nt intervals. (**C**) Infectious *in vitro* transcribed RNA was transfected into *Drosophila* S2 cells followed 48 h later by immunoblot detection of VP2. WT, wild-type CrPV infectious clone; ORF1/ORF2 STOP, mutant CrPV clones containing a stop codon in ORF1 or ORF2; ΔPKI, mutant IGR IRES containing mutations that disrupt PKI base pairing; CrPV–Cal, chimeric CrPV infectious clone containing an IGR IRES substituted by that of Caledonia virus. (**D**) CrPV and chimeric CrPV–Cal infection (MOI 5) in *Drosophila* S2 cells monitored by VP2 expression at the indicated hours post-infection (h.p.i.). (**E**) Viral yield of CrPV and chimeric CrPVx–Cal infection (MOI 0.1) at 12 and 24 h.p.i. using a log scale. Viral titers: FFU/μl. *P-value < 0.05; *n* = 3.

To determine whether type 6e IRESs can support infection, we generated a chimeric CrPV clone (‘CrPV–Cal’) (Figure 2A) containing the Caledonia IRES (Figure 2B) in place of the CrPV IGR IRES. *In vitro* transcribed RNA was transfected into *Drosophila* S2 cells and the expression of mature capsid protein VP2 was monitored by immunoblotting at 48 h post-transfection. VP2 is proteolytically processed from the P1 polyprotein, and its appearance is indicative of productive infection (38). VP2 expression was detected in cells transfected with wild-type CrPV mRNA but not with mutant mRNAs containing a stop codon in ORF1 or ORF2 (Figure 2C). Transfection of the chimeric CrPV–Cal RNA also resulted in detection of VP2, albeit at a lower intensity (Figure 2C). In contrast, a mutant chimeric CrPV–Cal clone containing a stop codon in ORF2 or containing substitutions that disrupt base pairing in PKI of the Caledonia IGR IRES did not lead to VP2 expression. These observations indicate that the Caledonia IGR IRES is active and can support infection in cells.

To further support these findings, the chimeric virus was propagated by passaging twice in S2 cells. RNA from the passaged virus was purified, amplified and sequenced, confirming the presence of the Caledonia IRES, thus demonstrating that the chimeric CrPV–Cal is stable (data not shown). We next infected naïve S2 cells (MOI 5) and monitored virus infection by VP2 expression at 2–8 hours post-infection (h.p.i.). VP2 expression in chimeric CrPV–Cal infection was lower at 2 and 4 h.p.i. than in CrPV infection (Figure 2D), suggesting that the Caledonia IRES is less active than the CrPV IRES. To determine whether there is an effect on virus replication, we infected S2 cells at a lower MOI (MOI 0.1) and then determined viral yield (Figure 2E). CrPV–Cal infected cells reproducibly showed lower viral yield at 12 and 24 h.p.i. than wild-type CrPV-infected cells, suggesting that the reduced replication of CrPV–Cal may be due to reduced translation from the Caledonia IRES. In summary, these results demonstrate that the Caledonia IGR IRES in a heterologous CrPV infectious clone can support virus infection.

### Functional validation of type 6e IRESs

The defining functional characteristic of IGR IRESs is their ability to bind productively to ribosomes in a factor-independent manner. Recruitment of ribosomes to type 6a and 6b IRESs depends on the interaction with the 40S subunit and, in primer extension inhibition (‘toeprinting’) assays, the intensity of the toeprints attributable to the bound 40S subunit is not enhanced nor does their position shift on joining with a 60S subunit [e.g. (13)]. On the other hand, type 6c and 6d IRESs bind directly to 80S ribosomes, but not stably to 40S subunits (5,20).

**Figure 3.**
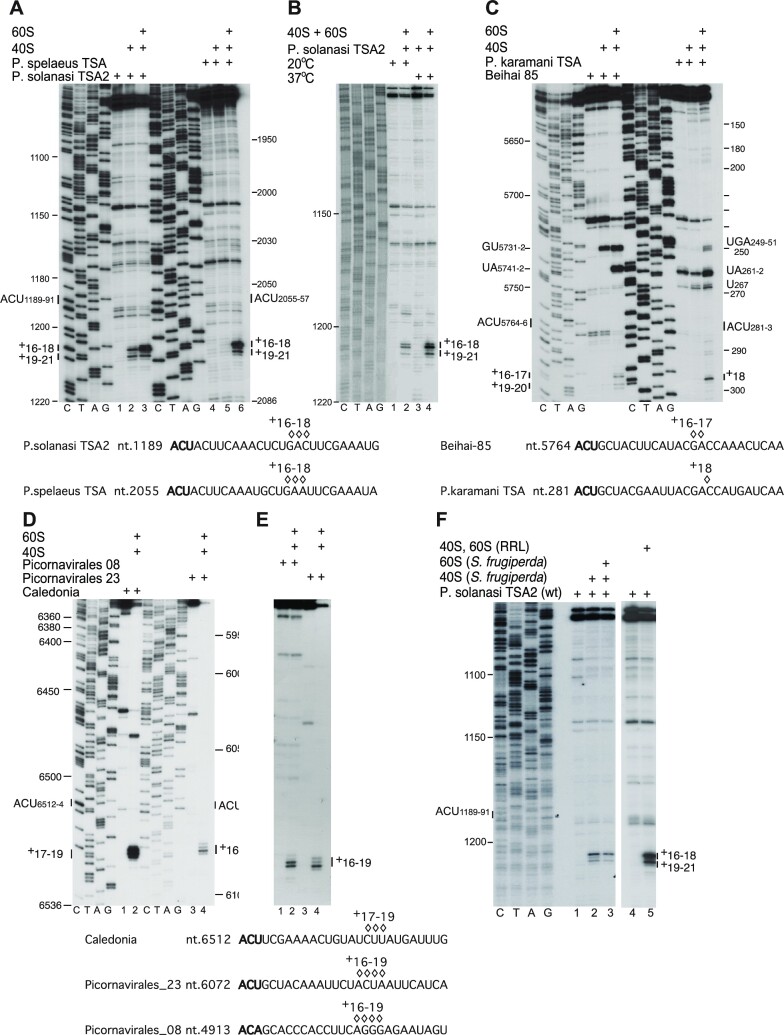
Factor-independent assembly of ribosomal complexes on type 6e IRESs. Binding of rabbit 40S subunits and assembly of 80S ribosomal complexes on (**A** and **B**) *P. spelaeus* TSA and *P. solanasi* TSA2 IRESs, (**C**) *P. karamani* and Beihai 85 IRESs, (**D**) Caledonia beadlet anemone dicistro-like virus 1 and picornavirales Q_sR_OV_023 (picornavirales 023) IRESs and (**E**) picornavirales N_OV_008 and Q_sR_OV_023 (picornavirales 08 and 023) IRESs in the presence of components of the translation apparatus (A–E) at 37°C and (B) also at 20°C, as indicated. (**F**) Binding of rabbit and insect (*S. frugiperda*) 40S subunits to the *P. solanasi* TSA2 IRES, assembly of 80S complexes and translocation at 37°C in the presence of the indicated components. The positions of toeprints relative to the +1 nucleotide of the triplet that mimics the initiation codon are indicated to the right and left of panels, as appropriate. A dideoxynucleotide sequence generated with the same primer was run in parallel on each gel (lanes C, T, A and G) to show the corresponding viral sequences. ORF2 sequences shown below each panel are annotated to indicate the positions of the strongest set of toeprints.

**Figure 4.**
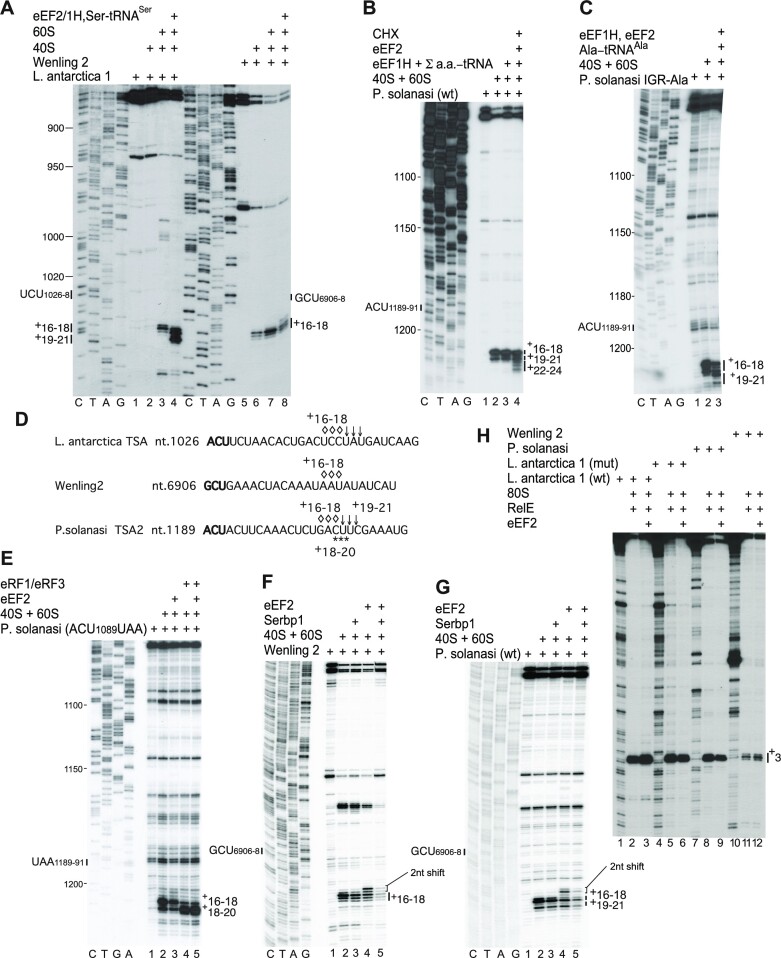
Factor-independent assembly of ribosomal complexes on type 6e IRESs, accessibility of the A site in IRES/80S ribosome complexes and eEF2-mediated translocation following recruitment of cognate aminoacyl-tRNA/eEF1H·GTP. (**A**) Binding of rabbit 40S subunits to Wenling 2 and *L. antarctica* TSA1 IRESs, assembly of 80S complexes and translocation in the presence of the indicated components. (**B** and **C**) eEF2-mediated ribosomal translocation in the presence of the indicated components on (B) wild-type and (C) ACU_1189–1191_ (Thr)→GCU (Ala) variant *P. solanasi* TSA2 IRESs. (**D**) ORF2 sequences, annotated to indicate the positions of toeprints in (A) and (B). (**E**) Toeprinting analysis of binding of eRF1 and eRF3 to 80S complexes assembled on *P. solanasi* IRES-STOP mRNA in the presence and absence of eEF2. (**F** and **G**) Influence of SERBP1 and eEF2 on the binding of ribosomes to (F) Wenling 2 and (G) *P. solanasi* TSA2 IRESs, assayed by toeprinting. The positions of toeprints of IRES-bound 80S complexes relative to the +1 nucleotide of the initiation codon (A) mimics UCU_1026–1028_ of the *L. antarctica* TSA1 IRES, (A and F) GCU_6906–6908_ of the Wenling 2 IGR IRES and (B–E, G) ACU_1189–1191_ of wild-type and GCU_1189–1191_ or UAA_1189–1191_ of mutant forms of the *P. solanasi* TSA2 IRES (A–C, E–G) before binding of factors and after (A–C) translocation or (E) binding of eRF1/eRF3 are indicated to the right and left of each panel, as appropriate. A 2 nt reverse shift in the toeprint induced by binding of eEF2 IRES/80S complexes is indicated in (F) and (G). A dideoxynucleotide sequence generated with the same primer was run in parallel on each gel (lanes C, T, A and G) to show the corresponding viral sequences. (**H**) Analysis of A-site accessibility in 80S ribosomes bound to Wenling 2, *P. solanasi* TSA2 and *L. antarctica* TSA1 wild-type and AUG_223–225_UAG mutant IRESs in the presence and absence of eEF2, assayed by mapping susceptibility to RelE cleavage. Positions of cleavages corresponding to IRES-bound ribosomal complexes are indicated to the right.

We tested the ability of type 6e IGRs to bind to mammalian and insect ribosomes and ribosomal subunits, using toeprinting to map the location of ribosomal complexes on mRNAs. All type 6e IRESs bound to rabbit 80S ribosomes, generally yielding toeprints +16–18 nt from the first (+1) nucleotide of the triplet in PKI that mimics the start codon base-paired to the anticodon stem–loop of tRNA (Figures 3 and 4). For some IRESs, including *P. solanasi* TSA2, *P. spelaeus* TSA (Figure 3A) and Beihai-85 (Figure 3B), a second weaker set of toeprints appeared +19–21 nt from the +1 nucleotide, 3 nt downstream of the first set. The observation of two sets of toeprints reflects binding of the PKI codon/anticodon mimic predominantly in the P site, but suggests that a fraction of the population may be in the E site. Partitioning of IGR IRESs between two states has been noted for CrPV, resulting from eEF2-mediated translocation from A to P sites and spontaneous back-translocation in the absence of an A-site ligand (19) and spontaneous factor-independent forward translocation from A to P sites (66). The appearance of two sets of toeprints on type 6e IRESs may not necessarily reflect spontaneous translocation, because they were observed with 40S subunits alone on the *P. solanasi* TSA2 IRES and could thus have arisen from direct binding of PKI to P or E sites.

The intensity of toeprints varied between viruses, indicative of differences in the level of binding of PKI in the P site: *P. solanasi* TSA2, *P. spelaeus* TSA, *L. antarctica* TSA1, Wenling 2 and Caledonia IGR IRESs bound strongly, and Beihai 85, *P. karamani* TSA, Wenling 2 and picornavirales N_OV_008 and Q_sR_OV_023 IRESs bound moderately (Figure 3A, C–E). Binding of 80S ribosomes to Beihai 85, Caledonia, *L. antarctica* TSA1 and *P. karamani* TSA IRESs also yielded toeprints in Stem 1 of PKI, suggestive of additional stabilizing interactions that involve this region of the IRES. Toeprints caused by binding of 40S subunits were generally very weak, except for *P. solanasi* TSA2 and Wenling 2 (Figures 3A and 4C). In contrast to the CrPV IRES, the intensity of toeprints on type 6e IRESs was strongly enhanced by joining of the 60S subunit; importantly, enhancement on the *P. solanasi* TSA2 IRES was restricted to the +16–18 toeprints and did not alter the intensity of the +19–21 nt toeprints. These observations suggest that the complex that yields the +16–18 toeprints contains the 40S subunit in an active conformation. In analogous experiments, the *P. solanasi* TSA2 IRES bound to insect 40S subunits and 80S ribosomes, yielding toeprints +16–18 nt and to a lesser extent +19–21 nt downstream of the codon–anticodon mimic (Figure 3F). Dicistroviruses generally infect poikilothermic organisms and may thus be exposed to low ambient temperatures, but although binding of ribosomes to the *P. solanasi* TSA2 IRES was weaker at 20°C than at 37°C, the pattern of toeprints did not differ (Figure 3B).

Type 6e IRESs therefore bind to eukaryotic ribosomes in a factor-independent manner, placing the PKI-adjacent coding region in the mRNA-binding cleft. They bind to mammalian and insect ribosomes in an equivalent manner, suggesting that they interact with conserved ribosomal elements.

### Accessibility of the initiation codon in the ribosomal A-site in binary IRES/80S complexes

The observations that ribosomes bind directly to type 6e IGR IRESs and undergo a programmed elongation step on addition of cognate Ser-tRNA^Ser^, eEF1H and eEF2 after binding to the *L. antarctica* TSA1 IRES (Figure 4A, lanes 3 and 4) but not after binding to the Wenling 2 IRES [which initiates at a GCU (Ala) codon] suggest that initiation on these IRESs broadly resembles initiation on type 6a and type 6c IRESs. However, details of the mechanisms of initiation on these two types of IRES differ in important respects. To place the initiation mechanism used by type 6e IRESs in the spectrum of mechanisms used by other IGR IRESs, we characterized individual steps in initiation on the *P. solanasi* TSA2 IRES.

Addition of a full set of aminoacylated tRNAs (Σaa-tRNA), eEF1H, eEF2 and cycloheximide enabled 80S ribosomes bound to this IRES to undergo a programmed cycle of elongation (Figure 4C, lane 4), indicating that it binds ribosomes in an active, elongation-competent conformation. The elongation step was recapitulated on an IRES variant with an ACU_1189–1191_ (Thr)→GCU (Ala) substitution by including eEF1H, eEF2, 40S and 60S subunits with cognate Ala-tRNA^Ala^ (Figure 4D, lanes 2 and 3). The position of the major set of toeprints derived from ribosomes bound to this IRES prior to addition of elongation factors suggested that ACU_1189–1191_ occupies the ribosomal A site and may be directly accessible to aminoacyl-tRNA without prior eEF2-mediated translocation of the IRES. The possibility that the ACU_1189–1191_ codon occupied the A site and was accessible to ligands was tested by assaying whether A-site ligands other than aminoacyl-tRNA could also bind to this codon in the absence of eEF2. The termination factors eRF1 and eRF3 bound to a mutant IRES in which ACU_1189–1191_ had been replaced by a UAA stop codon, indicating that this codon is accessible. Importantly, binding did not depend on eEF2 and was not affected by its inclusion in reactions (Figure 4E, lanes 4 and 5). The appearance of toeprints at positions +18–20 in the presence of eRF1/eRF3 is consistent with binding of eRF1 to the stop codon in the A site and consequent compaction of mRNA [e.g. (37)]. In contrast, eEF2-mediated translocation is required for eRF1/eRF3 to bind to an analogous stop codon variant of the CrPV IRES (19). The bacterial toxin RelE cleaves mRNA in the A site of ribosomal complexes (67). Whereas cleavage of the 80S ribosome-bound CrPV IRES occurs efficiently only in the presence of eEF2, reflecting the requirement for eEF2 for translocation of PKI out of the A-site (9), cleavage of ribosome-bound *P. solanasi* TSA2, Wenling 2 and Limacina 1 IRESs was eEF2 independent (Figure 4F).

Three different A-site ligands could therefore bind to ribosome-bound *P. solanasi* TSA2 and related IRESs in the absence of eEF2. Type 6e IRESs therefore resemble type 6c IRESs and differ from canonical type 6a and 6b IRESs in not requiring an eEF2-mediated pseudo-translocation step prior to decoding of the first codon.

A characteristic of type 6c and type 6d IRESs, which bind to the P site rather than the A site, is that binding is impaired by eEF2 and the Serpine mRNA-binding protein 1 (Serbp1), whereas binding of the type 6a CrPV IRES to the A site is resistant to inhibition (5,20). These proteins are associated with native ‘hibernating’ ribosomes (35,68). We recapitulated this inhibitory process *in vitro* using purified native eEF2 and recombinant Serbp1 and purified, salt-washed 40S and 60S ribosomal subunits as described previously (5,20). We determined that whereas Serbp1 alone modestly impaired ribosomal binding by *P. solanasi* TSA2 and Wenling 2 IRESs, inclusion of eEF2 with Serbp1 led to strong inhibition (Figure 4F, lanes 1–3, 5; Figure 4G, lanes 1–3, 5). Incubation of 80S/IRES complexes with eEF2 alone led to a –2 nt shift in the ribosomal toeprint, as previously noted for type 6c and 6d IRESs (5,20), probably due to changes in ribosomal conformation caused by eEF2-induced rotation of the ribosome (69,70).

### Validation of the predicted structure of the *P. solanasi* TSA2 IGR IRES

**Figure 5.**
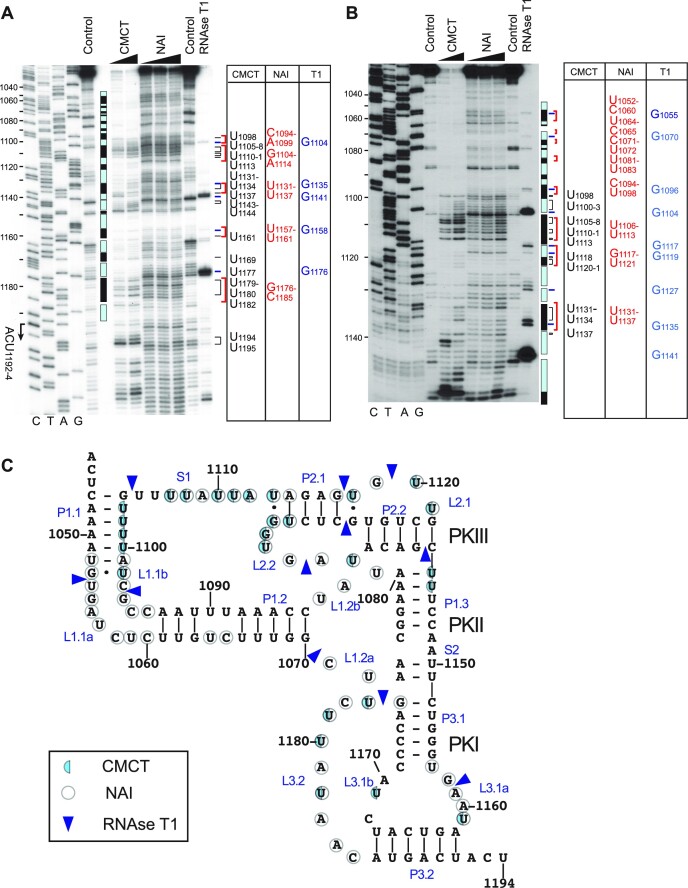
Structure of the *P. solanasi* TSA2 IRES. (**A** and **B**) Chemical (CMCT and NAI) and enzymatic (RNase T1) probing of the IRES. A dideoxynucleotide sequence generated with the same primer was run in parallel on both gels (lanes C, T, A and G) to show the *P. solanasi* TSA2 sequence. The positions of cleaved/modified nucleotides are indicated on the right of each panel, with a schematic representation of the IRES showing single-stranded loops as black rectangles and base-paired elements as light blue rectangles. The initiation codon mimic ACU_1189–1191_ is marked on the left. (**C**) Model of the tertiary structure of the *P. solanasi* TSA2 IRES, labeled to show nucleotides and pseudoknots, and indicating the positions of nucleotides modified by CMCT (shaded circles) and NAI (open circles) or cleaved by RNase T1 (arrowheads), based on data shown in (A) and (B).

The length (150 nt) and GC composition (39%) of the *P. solanasi* TSA2 IRES are representative of the type 6e group (GC content 33–46%), and it was therefore selected for structural validation. Probing was done using RNase T1 which is specific for unpaired G residues, CMCT which reacts with unpaired U and G residues (44), and NAI which forms 2′-*O*-adducts on single-stranded regions of RNA that can be detected by primer extension (45). The pattern of chemical modification (Figure 5) was consistent with the proposed model: strong modification occurred in S1, L2.2 and L3.2 loops, and more moderate modification was observed in other loops. Modification of the AU-rich helix P1.1 and the AU-rich segment of helix P1.3 suggest that these elements may be in equilibrium with partially unfolded forms. RNase T1 cleavage was apparent at the boundaries between helices and loops, and in loops L1.1a, L1.1b, L2.1a, L2.1b and L3.1a.

### Mapping the borders of the *P. solanasi* TSA2 IGR IRES

**Figure 6.**
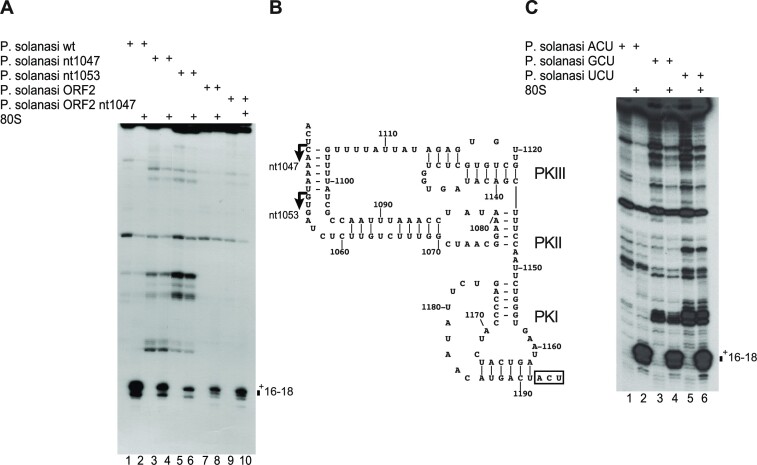
Mapping the borders of the *P. solanasi* TSA2 IRES. Activity of the *P. solanasi* TSA2 IRES variants (**A**) with 3′-terminal deletions in supporting accurate, factor-independent binding of 80S ribosomes and (**C**) substitutions in the first codon of ORF2. The positions of toeprints relative to the +1 nucleotide of the initiation codon mimic ACU_1189–1191_ are indicated to the right and left of each panel, as appropriate. A dideoxynucleotide sequence generated with the same primer was run in parallel on each gel (lanes C, T, A and G) to show the corresponding viral sequences. (**B**) Structural model of the *P. solanasi* TSA2 IRES, annotated to show the 5′ border of deletion mutants.

The borders of the IRES were established by deletion analysis and by substitution of the start codon. Toeprinting showed that the activity of mutant IRESs in promoting ribosomal recruitment to the translation start site was reduced but not abolished by truncation up to and including nucleotide 1047 at the 5′-border of helix P1.1 and was strongly reduced by truncation up to and including nucleotide 1052, which eliminates the first strand of this helix (Figure 6A, lanes 1–6; Figure 6B). Activity was also reduced, but not abolished, by replacement of nucleotides 1–60 of the ORF downstream of the initiation codon by a heterologous sequence in mRNAs containing the complete IGR IRES (Figure 6A, lanes 7 and 8) or lacking the first strand of helix P1.1 (Figure 6A, lanes 9 and 10). IRES-mediated ribosomal recruitment was unaffected by substitution of the ACU_1189–1191_ (Thr) initiation codon by GCU (Ala) or UCU (Ser) codons (Figure 6C), which correspond to codons that occur in other members of the Wenling class of IGR IRESs. The *P. solanasi* TSA2 IGR IRES is therefore 148 nt long, including the initiation codon.

### Validation of type 6e IGR IRES structure by mutational analysis

**Figure 7.**
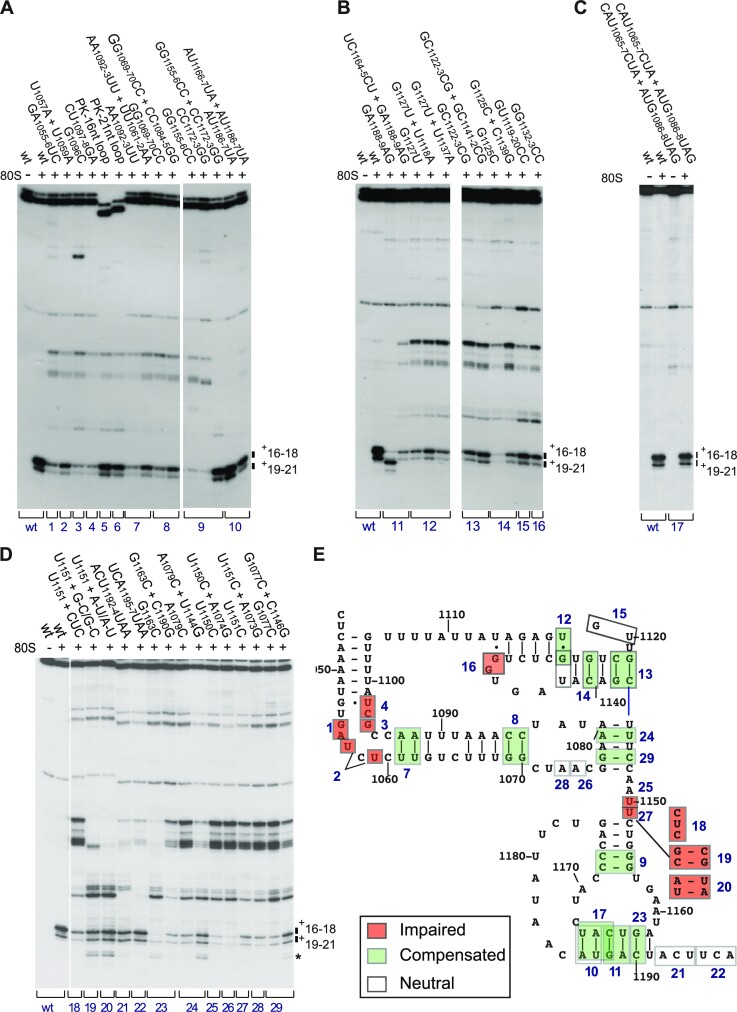
Mutational analysis of sequence and structural elements in the *P. solanasi* TSA2 IRES. (**A–D**) Toeprint analysis of assembly of 80S complexes on the wild-type (wt) and mutant variants of the *P. solanasi* TSA2 IRES. The positions of toeprints relative to the +1 nucleotide of the initiation codon mimic ACU_1189–11911_ are indicated to the right of each panel. Numbers below each panel refer to sites subjected to mutational analysis, as indicated in (**E**), which summarizes the effects of mutations on IRES function. ‘Compensated’ indicates sites at which a debilitating mutation could be compensated by second-site substitutions, and ‘Impaired’ indicates sites at which mutations that impair IRES function could not be compensated in this way. (A, B, D) Separation of lanes by white lines indicates that they were juxtaposed from the same gel.

The proposed structure of the type 6e *P. solanasi* TSA2 IGR IRES (Figure 5C) was consistent with the results of chemical and enzymatic probing. A complementary approach to test models of IRES structures is to determine if defects in function caused by destabilizing mutations could be suppressed by second-site substitutions that restore base pairing. Binding of mutants to ribosomes was assayed by toeprinting. Substitutions that destabilized helices P1.2 (sites 7 and 8), P1.3 (sites 24 and 29), P2.1 (site 12), P2.2 (site 13, 14), P3.1 (site 9) and P3.2 (sites 10, 11, 17, 23) (Figure 7E) strongly reduced or almost abrogated IRES function in binding to ribosomes (Figure 7A–D). Activity was partially or wholly restored by compensatory substitutions. The GC_1122–1123_CG substitutions in helix P2.2 (site 13) impaired IRES function, which was restored by compensatory GC_1141–1142_CG substitutions (Figure 7B). The AU_1186–1187_UA substitutions alone or with the AU_1166–1167_UA substitutions in P3.2 (site 10) had little effect (Figure 5A). The GA_1188–1189_AG substitutions in the center of P3.2 (site 11) led to a forward shift in the toeprint, probably due to formation of alternative base pairing for P3.2 that involves AGUCA_1162–1166_ and UGACU_1200–1204_ (Figure 7B, lanes 2 and 3). Importantly, compensatory substitutions in P3.2 restored initiation at the correct site (Figure 7B, lane 4) and, consistently, an IRES variant with AUG_1186–1188_UAG and CAU_1165–1167_CUA substitutions in P3.2 (site 17) had wild-type activity (Figure 7C). The loss of function caused by the G_1127_U substitution was restored by U_1118_A (site 12) but not by U_1137_A substitutions (Figure 7B), suggesting that U_1118_ and G_1127_ form a base pair, and thus that P2.1 and P2.2 both contain five base pairs. The complementary approaches of probing and mutational analysis thus yielded results that supported the proposed structural model.

### Functionally important sequence elements in the *P. solanasi* TSA2 IRES

**Figure 8.**
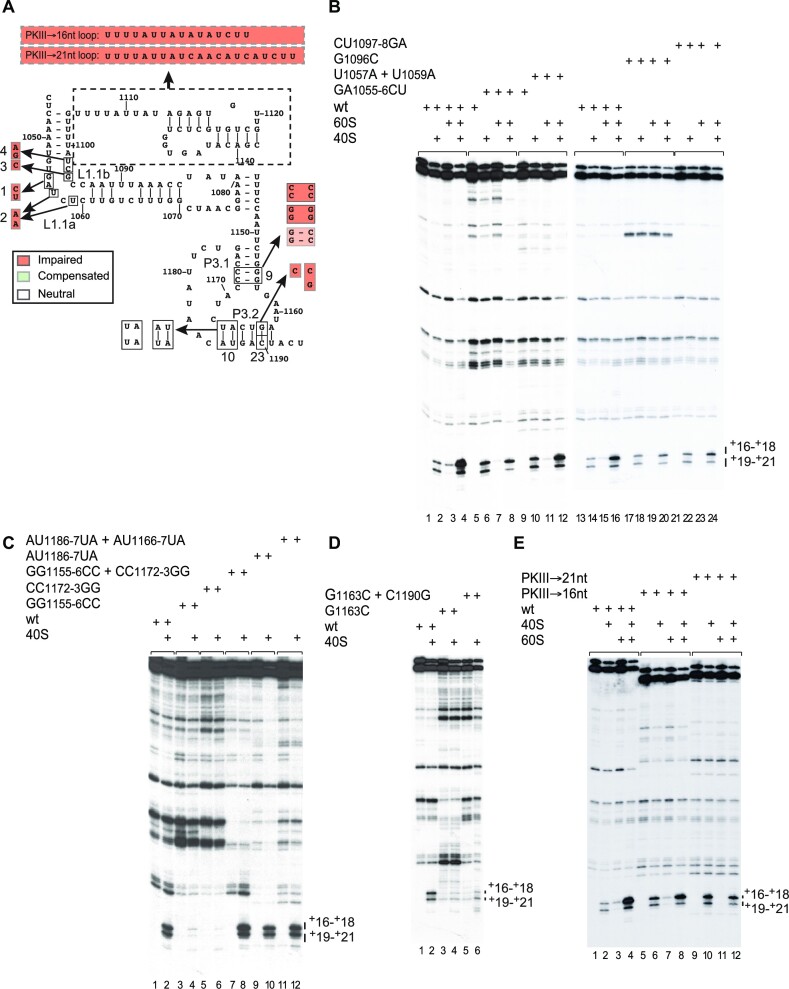
The influence of conserved structural elements in the *P. solanasi* TSA2 IRES on ribosome binding activity. (**A**) Model of the IRES, showing substitutions of nucleotides in PKI and the L1 loop, and nucleotides introduced to replace PKIII by unstructured loops. Sites of mutation are numbered as in Figure 7E. (B–E) Activity of IRES variants with substitutions (**B** and **C**) in PKI, (**D**) in the L1 loop and (**E**) of PKIII by single-stranded loops in factor-independent binding of 80S ribosomes and 40S subunits, assayed by toeprinting. The positions of toeprints relative to the +1 nucleotide of the initiation codon mimic ACU_1189–1191_ are indicated to the right of each panel, as appropriate.

Some motifs within IRESs have sequence-specific functions, whereas other elements have sequence-independent structural roles, for example to present specific motifs in an orientation that allows them to bind productively to IRES *trans*-acting factors (ITAFs) or components of the translation apparatus [e.g. (71–74)]. A critical step in initiation on IGR IRESs is their binding to the ribosome in a manner that places the first codon of ORF2 in the A site to be decoded. A panel of *P. solanasi* TSA2 IRES variants was used to identify elements that are required for functional interaction with 40S and 60S subunits (Figure 8).

Binding of 40S subunits to the IRES assayed by toeprinting was strongly impaired by disrupting the P3.1 helix in PKI (Figure 8C, lanes 3–6) but was relatively unaffected by a loss of base pairing in the portion of P3.2 distal to the start codon (Figure 8C, lanes 9 and 10). Loss of activity was fully restored by compensatory mutations (Figure 8C, lanes 7, 8, 11 and 12). In contrast, binding was strongly impaired by disruption of the 1163–1190 base pair and was only partially restored by a second-site substitution that restored base pairing (Figure 8D). These results show that the identity of nucleotide 1163, and not just its ability to base-pair with nucleotide 1190, is important for IRES function, and that PKI is responsible for binding of the IRES to the 40S subunit. The effects of these substitutions on binding to 40S subunits in each instance paralleled their influence on binding of 80S ribosomes (Figure 7D). The interaction with the 40S subunit therefore plays the dominant role in productive ribosomal binding by the IRES.

Loops L1.1a and L1.1b contain conserved motifs that resemble motifs in type 6a, 6b and 6c IRESs (Table 1). Substitutions in both loops of the *P. solanasi* TSA2 IRES impaired ribosomal binding: alterations in loop L1.1b had a greater effect on formation of IRES/80S complexes than substitutions in loop L1.1a (Figure 8A, B). GA_1055–1056_CU and U_1057_A/U_1059_A substitutions in loop L1.1a, and G_1096_C and CU_1097–1098_GA substitutions in loop L1.1b did not significantly affect binding of 40S subunits to the IRES (Figure 8B, lanes 2, 6 and 10), but inclusion of 60S subunits in these reactions strongly enhanced toeprints only in the case of the wild-type IRES, at a reduced level in the case of the U_1057_A/U_1059_A substitutions and not at all in the case of the other mutations (Figure 4A, lanes 2 and 3; Figure 8B, lanes 2, 4, 6, 8, 10 and 12). The appearance of a weak +16 nt toeprint on incubation of wild-type and some mutant mRNAs with 60S subunits (e.g. Figure 8B, lanes 3, 7 and 11) probably reflects the presence of contaminating 80S ribosomes in these preparations of 60S subunits. The L1.1 loop therefore does not influence the IRES/40S subunit interaction but is critical for productive binding of 60S subunits to *P. solanasi* TSA2 IRES/40S subunit complexes.

The compact, strongly conserved PKIII element is a distinctive feature of type 6e IRESs (Table 1), and mutational analysis (Figure 7B, E) indicated that it is an important determinant of ribosomal binding. The impairment of ribosomal binding caused by disruptive substitutions in P2.1 and P2.2 was suppressed by compensatory second-site substitutions that restored the potential for base pairing. Complete replacement of PKIII and the upstream single-stranded S1 element by 16 or 21 nt long single-stranded RNAs like that found at the equivalent position in the HalV IGR IRES (Figure 8A) did not prevent the *P. solanasi* TSA2 IRES from binding to 40S subunits (Figure 8E). However, relative to the wild-type IRES, the intensity of toeprints was either not enhanced or enhanced less strongly by inclusion of 60S subunits with 40S subunits and these mutant IRESs.

## DISCUSSION

### A conserved, structurally distinct subtype of IGR IRES

**Figure 9.**
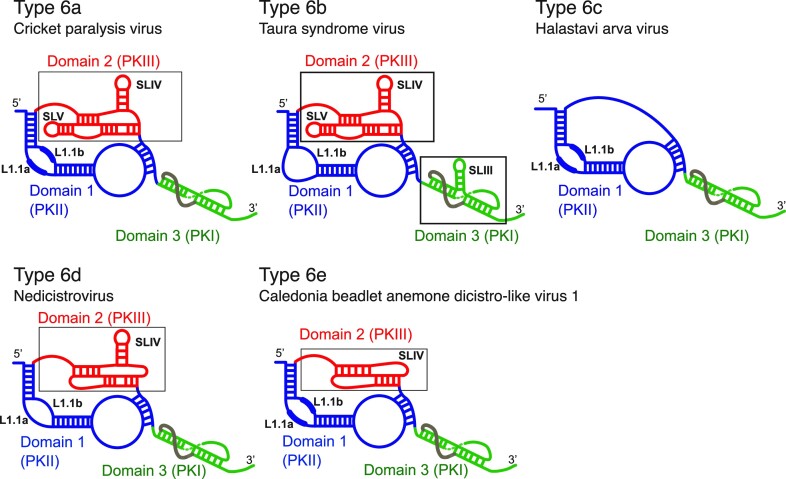
Schematic models of IGR IRES types 6a–6e. Models of (**A**) a type 6a IRES (e.g. cricket paralysis virus), (**B**) a type 6b IRES (e.g. Taura syndrome virus), (**C**) a type 6c IRES (e.g. Halastavi árva virus), (**D**) a type 6d IRES (e.g. Nedicistrovirus) and (**E**) a type 6e IRES (e.g. Caledonia beadlet anemone dicistro-like virus 1), labeled to indicate domain 1 (which includes PKII) colored blue, domain 2 (which includes PKIII) colored red and domain 3 (which includes PKI) colored green, stem–loops SLIII, SLIV and SLV, and the L1.1a and L1.1b loops (with conserved motifs in these loops indicated by thicker lines).

The Wenling group of IRESs identified here constitutes a distinct variant class of IGR IRES, designated as type 6e. They are a structurally conserved group, and the dicistroviruses in which they have been identified form a distinct clade. Type 6e IRESs share some characteristics with other IGR IRESs (Figure 9), including the use of a non-AUG codon for initiation, its location immediately downstream of a compact 3′-terminal pseudoknot (PKI) and the presence of an adjacent pseudoknot domain (PKII) that contains conserved L1.1 loop motifs that in other classes of IGR IRES interact with the L1 stalk of the 60S subunit [e.g. (5,9,11,12)]. These motifs are important for the function of the type 6e IRES in *P. solanasi* TSA2 (Figures 7A and 8B). Type 6e IRESs are 145–154 nt long, and thus intermediate in size between the recently identified type 6c IRESs epitomized by Halastavi árva virus (5) on the one hand and the type 6d IRESs (20) and the well-established type 6a and 6b IRESs on the other. Type 6c IRESs (5) and type 6e IRESs (Figure 1B) are slightly shorter than type 6a IRESs, due in part to the presence of shorter P1.2 and particularly P3.1 helices. The principal distinguishing structural characteristic of type 6e IRESs also reflects this difference in size: whereas type 6a and 6b IRESs contain a pseudoknot (PKIII) with two conserved stem–loops (SLIV and SLV) protruding from PKII that engage with ribosomal proteins in the head of the 40S subunit (7–9), type 6d IGR IRESs contain a PKIII domain that lacks an SLV-like stem–loop, type 6c IRESs have an extended single-stranded loop in place of PKIII and type 6e IRESs contain a compact, highly conserved H-type pseudoknot (PKIII) nested in PKII that lacks equivalents of both SLIV and SLV (Figure 9). The failure of some compensatory mutations that re-established the potential for base pairing to fully restore function (Figure 7B and E) may be because base pairs formed by different pairs of nucleotides are not structurally equivalent (75). These results, and the high level of sequence and not just structural conservation, could potentially reflect the importance of sequence-specific characteristics of RNA helices [such as bending, the width of the major groove and engagement in tertiary interactions (75,76)]. Replacement of PKIII by a loop did not affect binding of 40S subunits, but impaired 80S complex formation, suggesting either that PKIII contributes an interaction that promotes adoption of the IRES of an active conformation or that it stabilizes IRES/40S complexes in a state that permits binding of a 60S subunit.

### The mechanism of initiation on the type 6e IGR IRESs

The mechanism of translation initiation on type 6e IRESs more closely resembles initiation on type 6c IRES than on the better-characterized type 6a and 6b IRESs. Thus, type 6e IRESs all bind stably to the P site of 80S ribosomes but only a subset of them also binding directly to 40S subunits (Figures 4 and 5). Binding occurs in such a way that the ribosomal A site is vacant and can be decoded directly by a cognate aminoacyl-tRNA/eIF1A·GTP complex (Figure 4C, D). The origin of type 6e IRESs from viruses apparently infecting organisms from diverse phyla, the ability of the *P. solanasi* TSA2 IGR IRES to bind to ribosomes from organisms from different kingdoms (Figure 3F) and the activity of the Caledonia IGR IRES in *Drosophila* cells (Figure 2) suggest that type 6e IRESs interact with broadly conserved elements of the eukaryotic ribosome. The position of PKI adjacent to the first triplet to be decoded and the fact that translocation is not required for this decoding step to occur suggest that PKI binds directly in the P site. Type 6e IRESs therefore differ from type 6a and type 6b IRESs, which bind in the A site and must be translocated to the P site in an eEF2-mediated process before decoding can occur [e.g. (9,19)]. Instead, type 6e IRESs bind to ribosomes in a way that mimics the post-translocation state, and they therefore use a more streamlined initiation mechanism than type 6a and 6b IRESs.

The difference between type 6a and type 6b IRESs, which first occupy the A site, and type 6c and type 6e IRESs, which bind directly to the P site, probably reflects the presence of SLIV and SLV only in the former. Although these elements are important for type 6a IRES function in cell-free extracts (17,77,78), they are not essential for viral translation during infection or, indeed, for viral infectivity (79). The change in the translation apparatus induced by infection that enables IGR IRESs lacking SLIV and/or SLV to function effectively has not been determined. However, the observation that IGR IRES function is activated during viral infection (80) could be accounted for by modification and consequent activation of ribosomes, or by dissociation of an inhibitor. A variety of factors sequester eukaryotic 80S ribosomes in an inactive state, including Serbp1 and eEF2, CCDC124 and its orthologs, and the interferon-related developmental regulator 2, which binds to the P site and extends through the E site to the mRNA channel exit (68,81). Ribosomal association of these factors is regulated by signaling pathways and could thus change dynamically during infection. Serbp1 and eEF2 inhibit ribosomal association of type 6c, 6d and 6e IRESs that bind to the P site, but not of type 6a IRESs that bind to the A site (5,20; Figure 4F, G), and they could thus selectively impair initiation mediated by IGR IRESs that lack SLIV and SLV. In this scenario, acquisition of SLIV/SLV equivalents by an IGR IRES leads to a gain in function.

### Evolution of IGR IRESs

The recent identification of type 6c IGR IRESs (5), type 6d IRESs (20) and type 6e IRESs, and the exponential growth in the number of unclassified dicistrovirus sequences together suggest that it is likely that additional structurally divergent classes of IGR IRESs will be identified. It is accordingly now reasonable to speculate on the evolutionary processes that have led to the appearance of these complex RNA structures. The model for IRES evolution by accretion of functional domains (82), akin to the model for evolution of rRNA (83) is, for example, consistent with the occurrence in type 6b IRESs of the branching SLIII stem–loop protruding from PKI (Figure 9) which is coaxially stacked on this domain, mimicking the elbow of tRNA and, like it, positioned to be able to interact with the A-site finger of rRNA in the 60S subunit, probably contributing to translocation of PKI from A to P sites (10–12). More generally, the distributed nature of functional elements in IGR IRESs, such as motifs in PKII that bind to the L1 stalk, and elements in PKI that determine binding in the P site, correctly orient the proximal coding region in the mRNA-binding cleft of the ribosome and establish the reading frame would be consistent with an evolutionary trajectory in which a primordial IGR IRES might consist only of the tRNA/mRNA-mimicking PKI, which would initially bind to ribosomes by virtue of its proximity to the ORF1 termination codon. Selection of sequences that could form a PKI-like pseudoknot would be followed by fusion with an extended PKII-like domain that contains L1 stalk-binding motifs. Acquisition of an H-type pseudoknot of the type identified here either by recombination or by non-templated addition of nucleotides followed by selection of pseudoknot-forming elements could then be followed by expansion of loop sequences in the pseudoknot to form stem–loop structures. The likelihood that additional classes of IGR IRESs remain to be identified suggests that further analysis of dicistrovirus sequences will provide more detailed insights into their evolution.

## Supplementary Material

gkad569_Supplemental_File

## Data Availability

All relevant data are available in the manuscript and the supplementary materials.
