# Supplementary material for: The structure and mechanism of action of a distinct class of dicistrovirus intergenic region IRESs
Source: Nucleic Acids Res. 2023 Jul 10;51(17):9294–313. doi: 10.1093/nar/gkad569 (PMC10516663; doi:10.1093/nar/gkad569)
Supplement: gkad569_Supplemental_File [file gkad569_supplemental_file.pdf]

Supplementary Table 1. Amino acid sequence identity in the 3C protease/3D polymerase segment encoded by ORF1 of Wenling picorna-like virus 2 and related viruses.

	1	2	3	4	5	6	7	8	9	10	11	12	13	14	15	16	17	18	19	20	21	22
1: HalV	**100**	**33.2**	**33.4**	**32.7**	23.6	25.9	27.1	27.3	28.2	27.4	26.0	26.2	26.2	26.3	27.1	26.8	27.1	27.3	26.8	27.0	27.4	27.6
2: Shahe		**100**	**51.2**	**52.6**	23.8	23.1	26.1	25.7	26.2	25.1	24.9	23.6	23.6	25.2	23.2	25.0	23.7	25.0	24.9	24.8	24.5	24.3
3: Kuiper			**100**	**71.2**	23.5	23.3	27.6	26.0	26.7	24.9	27.0	24.6	25.3	26.7	26.0	26.3	25.4	26.0	26.4	25.4	25.7	25.3
4: CPLV14				**100**	24.0	24.6	27.3	25.7	25.7	25.3	26.4	24.3	26.2	26.4	26.7	26.2	26.4	26.8	26.8	26.5	25.7	26.0
5: Triatoma					100	27.0	28.9	26.2	27.1	26.2	26.3	25.0	25.6	27.7	25.2	25.5	25.3	26.3	25.7	25.4	25.0	25.3
6: CrPV						100	34.5	31.4	30.0	30.9	31.3	29.7	30.0	30.8	30.6	32.1	31.9	32.1	30.4	31.6	30.9	30.1
7: ABPV							100	32.7	29.9	30.8	28.9	28.7	29.3	31.9	31.0	32.0	31.2	31.9	31.8	32.2	31.9	31.1
8: TSV								100	31.1	32.6	32.1	31.5	30.7	32.7	31.1	31.7	31.5	32.5	31.9	31.6	32.1	31.9
9: Bivalve-G1									**100**	**50.2**	**47.5**	**51.5**	**49.5**	**53.9**	**52.9**	**51.7**	**52.0**	**52.2**	**52.6**	**52.1**	**51.7**	**51.1**
10: Beihai 85										**100**	**50.0**	**53.8**	**54.9**	**56.8**	**56.6**	**55.5**	**56.8**	**58.5**	**57.2**	**56.9**	**57.3**	**56.8**
11: Caledonia											**100**	**54.7**	**56.0**	**56.7**	**58.4**	**57.7**	**58.3**	**60.3**	**59.0**	**59.4**	**58.6**	**58.1**
12: Picorna_008												**100**	**59.8**	**61.0**	**60.5**	**61.1**	**61.1**	**62.6**	**61.6**	**60.9**	**61.8**	**61.4**
13: Wenling2													**100**	**65.8**	**68.0**	**67.5**	**67.6**	**69.1**	**68.3**	**69.0**	**69.0**	**68.6**
14: HPLV11														**100**	**67.8**	**70.6**	**73.2**	**74.1**	**73.4**	**73.1**	**72.2**	**71.5**
15: *H.dujardinii* TSA															**100**	**72.6**	**75.6**	**74.2**	**75.5**	**75.9**	**75.7**	**74.7**
16: *O. vulgaris* TSA																**100**	**80.8**	**81.0**	**81.8**	**83.2**	**81.2**	**80.9**
17: Wenling3																	**100**	**84.8**	**85.9**	**86.6**	**84.8**	**84.8**
18: Picorna_023																		**100**	**86.7**	**86.6**	**84.8**	**84.4**
19: Picorna_042																			**100**	**87.3**	**86.4**	**86.2**
20: Halhan1																				**100**	**87.8**	**87.7**
21: Bivalve-G5																					**100**	**95.9**
22: G5_Abbotsbury																						**100**

Percentage sequence identity determined by alignment using Clustal Omega of 3CD sequences from (1) Halastavi árva virus (HalV), (2) Shahe arthropod virus 1, (3) Kuiper virus, (4) Changjiang picorna-like virus 14 (CPLV14), representative members of the genera *Cripavirus* ((5) Cricket paralysis virus; CrPV), *Triatovirus* ((6) Triatoma virus), two clades of the genus *Aparavirus* ((7) Acute bee paralysis virus (ABPV) and (8) Taura syndrome virus (TSV) of *Dicistroviridae* and (9) bivalve RNA virus G1, (10) Beihai picorna-like virus 85, (11) Caledonia beadlet anemone dicistro-like virus 1, (13) Wenling picorna-like virus 2, (14) *Picornavirales* sp. isolate HPLV-11, (15) *Halisarcula dujardinii* TSA, (16) *Octopus vulgaris* TSA, (17) Wenling crustacean virus 3, (18) Picornavirales Q_sR_OV_023, (19) Picornavirales Q_sR_OV_042, (20) Halhan virus 1, (21) bivalve RNA virus G5 and (22) bivalve RNA virus G5 strain Abbotsbury/A/2016.

Sequence identity between 3CD moieties encoded by members of the proposed Halárvirus clade is indicated by bold text and yellow shading. Sequence identity between 3CD moieties encoded by members of the Wenling group is indicated by bold text and light blue shading.

Supplemental Table 2. Amino acid sequence identity in the capsid protein precursor encoded by ORF2 of Wenling picorna-like virus 2 and related viruses.

	1	2	3	4	5	6	7	8	9	10	11	12	13	14	15	16	17	18	19	20	21	22	23	24	25	26
1: HalV	**100**	**46.4**	23.5	21.3	20.7	20.3	19.2	19.4	19.2	20.8	20.5	20.2	20.0	19.5	20.3	20.2	19.7	19.6	20.4	19.5	20.6	19.5	20.1	19.4	19.8	20.1
2: Kuiper		**100**	24.9	23.3	21.0	21.8	18.8	19.6	21.4	20.1	19.9	20.0	19.6	19.8	20.5	19.9	18.9	20.6	20.4	19.4	19.9	20.4	20.0	20.2	20.6	20.5
3: CrPV			100	27.3	21.7	19.4	22.8	24.2	21.7	22.4	22.0	22.1	23.2	22.7	23.2	22.7	22.6	22.5	21.4	22.4	21.9	22.7	22.1	23.8	23.4	23.9
4: Triatoma				100	23.1	21.6	22.9	21.8	22.1	22.0	22.0	23.3	21.5	22.6	23.0	20.6	21.7	21.3	21.3	22.3	21.4	22.0	21.6	22.4	21.8	22.1
5: ABPV					100	23.3	22.2	23.7	24.5	24.4	23.3	22.1	22.7	24.0	23.7	21.7	23.8	22.8	22.3	23.2	22.9	24.4	23.2	23.7	23.2	23.1
6: TSV						100	25.6	25.5	25.4	25.3	24.4	26.3	24.9	24.3	26.9	25.1	24.7	25.4	25.5	25.2	25.8	26.1	25.7	26.2	25.9	26.4
7: Bivalve-G1							**100**	**46.2**	**48.0**	**48.3**	**48.5**	**49.0**	**46.8**	**45.6**	**48.8**	**48.2**	**48.7**	**46.4**	**47.8**	**48.0**	**48.5**	**48.6**	**48.5**	**48.9**	**49.5**	**49.9**
8: Caledonia								**100**	**53.2**	**54.4**	**52.6**	**57.9**	**51.3**	**53.7**	**54.7**	**55.4**	**55.4**	**55.5**	**54.4**	**55.1**	**55.3**	**56.2**	**55.7**	**56.1**	**55.8**	**55.4**
9: *P.karamani* TSA									**100**	**65.3**	**63.3**	**59.8**	**55.9**	**58.3**	**60.0**	**58.3**	**61.5**	**59.0**	**61.2**	**63.4**	**61.7**	**62.7**	**61.5**	**63.4**	**63.0**	**63.4**
10: *P.solanasi* TSA2										**100**	**82.5**	**59.2**	**57.1**	**56.1**	**57.8**	**58.1**	**61.2**	**57.9**	**60.4**	**59.9**	**60.7**	**62.1**	**60.3**	**62.5**	**61.6**	**61.8**
11: *P.spelaeus* TSA											**100**	**57.4**	**55.7**	**55.0**	**57.4**	**57.0**	**59.6**	**57.2**	**58.3**	**58.2**	**60.5**	**61.0**	**59.3**	**60.3**	**59.8**	**60.3**
12: Picorna_008												**100**	**57.2**	**60.7**	**62.2**	**61.1**	**61.5**	**60.3**	**61.0**	**61.4**	**62.4**	**62.1**	**61.3**	**61.8**	**61.9**	**62.1**
13: HPLV11													**100**	**58.0**	**55.8**	**58.4**	**56.8**	**56.1**	**59.7**	**58.4**	**58.8**	**59.4**	**59.1**	**59.0**	**59.4**	**59.3**
14: Beihai85														**100**	**58.5**	**61.0**	**60.4**	**59.9**	**60.5**	**62.2**	**63.1**	**64.0**	**62.6**	**63.3**	**62.1**	**62.6**
15: Wenling2															**100**	**62.2**	**63.2**	**61.4**	**61.4**	**63.3**	**65.7**	**64.3**	**63.3**	**64.4**	**64.4**	**64.4**
16: Limacina TSA2																**100**	**64.4**	**62.7**	**65.2**	**65.2**	**64.9**	**66.6**	**66.7**	**67.3**	**66.2**	**65.3**
17. *H.dujardinii* TSA																	**100**	**65.2**	**65.5**	**66.2**	**67.8**	**70.0**	**67.2**	**71.8**	**68.9**	**69.0**
18:*P.semisulcatus*																		**100**	**67.8**	**68.1**	**68.9**	**70.2**	**70.5**	**72.6**	**71.5**	**71.1**
19: Picorna_042																			**100**	**70.5**	**72.4**	**72.4**	**73.1**	**72.8**	**73.7**	**73.0**
20: Wenling3																				**100**	**73.2**	**73.5**	**74.5**	**77.2**	**76.6**	**75.9**
21: Picorna_023																					**100**	**78.4**	**77.4**	**77.1**	**78.3**	**77.2**
22: *O.vulgaris* TSA																						**100**	**81.8**	**81.4**	**79.1**	**77.2**
23: halhan																							**100**	**83.1**	**79.8**	**78.1**
24: *O.oratoria* TSA																								**100**	**85.5**	**83.2**
25: Bivalve-G5																									**100**	**92.5**
26: G5Abbotsbury																										**100**

Percentage sequence identity determined by alignment using Clustal Omega of ORF2 sequences from (1) Halastavi árva virus (HalV), (2) Kuiper virus, (3) Cricket paralysis virus (CrPV) of the genus *Cripavirus*, (4) Triatoma virus of the genus *Triatovirus*, (5) Acute bee paralysis virus (ABPV) and (6) Taura syndrome virus (TSV), representing of clades of the genus *Aparavirus Dicistroviridae*, (7) bivalve RNA virus G1, (8) Caledonia beadlet anemone dicistro-like virus 1, (9) *Proasellus karamani* TSA, (10) *P. solanasi* TSA2, (11) *P. spelaeus* TSA, (12) *Picornavirales* Q_sR_OV_008, (13) *Picornavirales* sp. isolate HPLV-11, (14) Beihai picorna-like virus 85, (15) Wenling picorna-like virus 2, (16) *Limacina antarctica* TSA2, (17) *Halisarcula dujardinii* TSA, (18) *Penaeus semisulcatus* TSA, (19) Picornavirales Q_sR_OV_042, (20) Wenling crustacean virus 3, (21) Picornavirales Q_sR_OV_023, (22) *Octopus vulgaris* TSA, (23) halhan virus 1, (24) *Oratosquilla oratoria* TSA, (25) bivalve RNA virus G5, and (26) bivalve RNA virus G5 strain Abbotsbury/A/2016. Sequence identity between ORF2 moieties encoded by members of the proposed Halárvirus clade is indicated by bold text and yellow shading. Sequence identity between ORF2 moieties encoded by members of the Wenling group is indicated by bold text and light blue shading.

Supplementary Table 3. Nucleotide identity amongst type 6e IGR IRESs.

	1	2	3	4	5	6	7	8	9	10	11	12	13	14	15	16	17	18	19	20	21	22	23	24	25	26
1: BivalveG1	100	58	66	64	59	63	60	58	66	63	65	62	66	68	65	65	65	65	65	64	66	64	69	63	65	64
2: Limacina2		100	64	59	62	63	62	60	61	60	58	57	58	58	59	60	61	61	61	60	58	57	67	66	62	61
3: Behai85			100	62	65	69	66	64	72	69	64	60	67	65	63	64	68	66	67	64	66	63	70	63	64	64
4: L. albus TSA				100	79	81	74	72	74	82	73	72	77	74	75	77	74	77	78	76	74	78	74	72	73	73
5: P.Karamani TSA					100	84	81	71	66	75	70	67	70	70	72	71	71	72	76	72	70	72	71	70	72	71
6: P.Solanasi TSA2						100	89	70	74	79	70	67	71	70	65	68	66	68	70	67	68	73	72	73	73	73
7: P.Spelaeus TSA							100	71	72	78	68	67	66	69	67	68	66	68	71	70	69	71	72	74	74	75
8: A.viridis TSA								100	73	79	76	68	72	74	71	71	70	72	74	71	74	77	74	74	74	75
9: Caledonia									100	78	71	70	74	73	70	72	71	72	72	71	74	72	71	71	69	70
10: Wenling-2										100	80	77	80	79	76	82	76	78	80	77	77	77	81	77	77	77
11: Picornav.08											100	70	75	74	74	74	72	75	80	73	71	71	78	68	71	70
12: HPLV11												100	75	78	79	78	79	80	80	81	82	80	81	74	74	75
13: Bivalve G5													100	95	79	83	79	81	82	80	84	75	77	74	76	76
14: G5Abbotsbury														100	80	84	82	83	85	83	88	77	78	74	76	75
15: Wenling-3															100	85	85	85	87	89	89	71	75	73	72	73
16: P.semisulcatus TSA																100	89	92	88	88	86	73	80	74	75	75
17: O. vulgaris TSA																	100	95	87	88	88	72	79	72	73	74
18: Loxomitra																		100	90	89	88	73	81	73	76	77
19: Halhan																			100	91	89	77	81	75	79	79
20: O.oratoria TSA																				100	91	73	76	74	78	78
21: Picornav.23																					100	75	79	75	75	76
22: E.giganteus																						100	80	78	80	81
23: Picornav.42																							100	80	79	80
24: Limacina TSA																								100	89	90
25: H.dujardinii TSA																									100	99
26: Terminoflustra TSA																										100

Percentage sequence identity was determined by alignment of IGR IRES sequences using Clustal Omega.
